# Clinical Audit: Promotion of Activities to Help Wellbeing in the Management of Older Adults With Depression in a Local NHS Community Mental Health Older People’s Service

**DOI:** 10.1192/bjo.2026.11832

**Published:** 2026-06-30

**Authors:** Fraser Ritchie, Manimegalai Chinnasamy

**Affiliations:** Leeds and York Partnership NHS Foundation Trust, Leeds, United Kingdom

## Abstract

**Aims:**

This audit aimed to determine clinician adherence in Leeds and York Partnership NHS Foundation Trust’s (LYPFT) Older People’s Service (OPS) North as part of the Community Mental Health Team (CMHT), for the NICE guidance regarding:

1. Provision of advice for people with depression for regular physical activity to enhance patients’ sense of wellbeing with potential for added benefits for outdoor activity.

2. Advising people with depression that maintaining a healthy lifestyle by, for example, eating a healthy diet, avoiding excess alcohol, and maintaining a healthy sleeping pattern, can also improve their sense of wellbeing.

**Methods:**

The first 200 consecutive contacts by service users with the service from 1^st^ July 2024 onwards were analysed. Seventy-six separate service users were identified. After excluding those without a depressive disorder noted, frail service users, and those who were exclusively seen by a psychologist within the defined study period – first contact from 1^st^ July 2024 up to one month later – 31 service users formed the final sample. Records were then searched to identify clinicians’ interactions with the service user and whether there was evidence of following the NICE guidelines for the provision of advice to improve patients’ sense of wellbeing.

**Results:**

Of the 31 patients in the final analysis, the NICE guidelines regarding the provision of advice for regular physical activity to enhance a sense of wellbeing were followed in 35% of cases (11 service users) with there being no documented evidence of such advice for the remaining 20 (65%) service users. Eighteen service users (58%) had no documented evidence of the provision of advice for maintaining a healthy lifestyle but 13 service users (42%) did. When analysing by staff group, compared to Care Coordinators and Health Support Workers, Doctors performed worst with regards to evidence of providing advice for behaviours to enhance wellbeing.

**Conclusion:**

This audit demonstrated that documentation of LYPFT CMHT OPS North clinicians’ advice to patients with depression that wellbeing can be improved by engaging in regular physical activity, and by maintaining healthy lifestyle habits is absent in the majority of cases (65% and 58% respectively). Following the audit, an action plan was created to improve adherence to the guidelines. This included staff education and creation of a patient information leaflet. A re-audit is planned to analyse whether the action plan has led to improvement.

